# Supplementary material for: Are carbon emissions trading and green financial instruments synergistic? -Comprehensive quantitative research based on content analysis
Source: PLoS One. 2024 Mar 7;19(3):e0298601. doi: 10.1371/journal.pone.0298601 (PMC10919601; doi:10.1371/journal.pone.0298601)
Supplement: S1 Data — (ZIP) [file pone.0298601.s001.zip › Data/地级市Z_F工作报告.zip!/╜¡╦╒/╤∩╓▌╩╨/└┤╫╘╣1⁄2╓┌║┼í╛┬φ┐╦╩2╛▌═°í┐_2010.docx]

各位代表：

现在，我代表扬州市人民政府，向大会作工作报告，请予审议，并请市政协各位委员和其他列席人员提出意见。

2009年政府工作回顾

2009年是极不平凡的一年。面对国际金融危机的严峻形势，我们在中共扬州市委的领导下，以科学发展观为统领，认真贯彻落实中央和省一系列决策部署，坚定信心，危中寻机，迎难而上，保增长、保民生、保稳定，基本完成市六届人大二次会议确定的目标任务。预计全年地区生产总值1805亿元，增长13.6%；财政总收入310.09亿元，其中一般预算收入128.08亿元，分别增长16.5%和22.2%；全社会固定资产投资1063.9亿元，增长33.8%；城市居民人均可支配收入19500元，农民人均纯收入8240元，分别增长12%和10.6%。

一、经济发展企稳向好

工业持续回升。规模以上工业实现产值4460亿元、利税325亿元，分别增长26.4%和21%。全市规模以上工业增加值1131亿元、高新技术产业产值1361亿元，江都规模以上工业产值1113亿元、邗江全部工业产值1130亿元，实现“四个千亿元”突破。石油化工、汽车船舶和机械装备三大主导产业产值3078.4亿元，增长28.9%；新能源、新光源、新材料“三新”产业产值344.7亿元，增长41.6%，占规模以上工业产值比重达7.7%。完成工业投入903亿元，增长30%。投资亿元以上竣工或部分投产项目140项，其中10亿元以上10项。新增规模以上企业423家，累计3446家。新创中国驰名商标7个。单位地区生产总值综合能耗下降4.6%。建筑业总产值1209亿元，增长15%；获鲁班奖3项。

服务业加快发展。实现增加值655.2亿元，增长14.5%，占地区生产总值比重36.3%。江苏信息服务产业基地（扬州）签约入驻企业40家。软件与信息服务业销售收入65亿元，增长18%。石化、港口、公铁水等重点物流园区加快建设。承办了第44届全国旅交会和第19届中国厨师节。蜀冈—瘦西湖风景名胜区获“国家文化旅游示范区”和“全国文明风景旅游区”称号。来扬游客2270万人次，旅游总收入245亿元，均增长20%以上。华夏银行扬州支行、邗江民泰村镇银行正式挂牌营业。金融机构年末存贷款余额为2067亿元和1212.8亿元，分别增长33.2%、36.4%。

农业稳步增长。全年粮食总产282万吨，创历史新高，连续6年丰收。新增高效农（渔）业面积54.4万亩、设施农业6.2万亩、适度规模经营34万亩。海峡两岸（扬州）农业合作试验区建设加快推进。农业利用外资2.16亿美元，利用民资58.4亿元，新增项目800个。新建农村“三大合作”组织815个。80家市级以上农业龙头企业实现销售176亿元，增长20.3%。农业、水利及综合开发等争取省以上投资10.9亿元。新创“三品”品牌67个。国家有机食品质量监督检验中心落户宝应。土地复垦与综合整治新增耕地2.54万亩。粮食流通、供销、农机、气象等工作得到加强。

扩内需成效显现。获批中央扩内需项目175个，计划投资22.7亿元，争取中央资金4.6亿元。投资亿元以上、当年投入5000万元以上项目252个，比上年增加37个。家电汽车下乡、家电以旧换新和农机具财政补贴等各项刺激消费政策得到较好落实。“万村千乡”市场工程持续推进。社会消费品零售总额619亿元，增长18.7%。全市房地产开发投资129.5亿元，竣工商品房505.9万平方米。市区商品房销售面积235.2万平方米，增长73.7%。

二、开放创新深入推进

科技创新能力进一步提高。新认定国家高新技术企业60家。全年高新技术产业产值增长57.2%，占规模以上工业产值比重30.5%，比上年提高5.9个百分点。获批省级以上科技计划项目456项，争取上级科技资金1.8亿元。研发经费支出占地区生产总值比重1.5%。成功举办科技创新成果展暨洽谈会和上海、南京、长春“科技创新·产业合作”推介会，签订产学研合作项目227个。新增省级以上“两站两中心”29家，各县（市、区）均建立省级创业服务中心。引进高层次领军人才153人、产业发展急需专业技术人才1384人。国家光电重点实验室、国家洗漱用品检测中心投入运行。扬州被列为国家“十城万盏”半导体照明应用工程试点城市、绿色新能源特色产业基地。荣获“全国科技进步先进市”称号。

改革开放不断深化。开展了境内外系列招商引资活动，成功举办“烟花三月”国际经贸旅游节。协议利用外资56.01亿美元、注册外资实际到账22.66亿美元，分别增长11.8%和31.7%；新批注册外资1000万美元以上项目195个。新增民营企业注册资本金543.1亿元，增长35.1%；新开工投入5000万元以上民资项目365个。外贸出口40.1亿美元。外经营业额2.2亿美元。扬柴与潍柴合作重组。市开发区开发总公司和城建控股公司企业债券成功发行。扬农化工实现再融资，3家企业上市材料报证监会审批。新增35家担保公司和6家农村小额贷款公司、6家农村资金互助合作组织。完成10个乡镇集体林权制度改革。

园区建设取得新进展。市开发区升级为国家级经济技术开发区，光电产业园被列为省十大创新型园区。“八区二园”新增开发面积8平方公里，实现业务总收入3815亿元。一般预算收入59亿元、注册外资实际到账18.25亿美元、新增民营企业注册资本金378亿元，分别占全市总量的46%、81%和70%。37个乡镇工业集中区营业收入增长35%。

三、城乡建设迈开新步伐

重大基础设施建设加快推进。江海高速扬州段、沪陕高速江六段、京杭运河扬州段“三改二”、邵伯和施桥三线船闸扩容、新民滩特大桥、乌塔沟分洪道等工程加快建设。宁启铁路复线及电气化改造、扬州港5号泊位开工建设。苏中江都机场项目已经国务院常务会议通过。连淮扬镇铁路、淮河入江水道整治和500千伏扬州西输变电工程等项目的前期工作取得积极进展。

城乡统筹稳步发展。出台了《关于推进市区率先统筹城乡发展的实施意见》。完成“强县强镇、三年倍增”目标任务。农村“十大工程”、“新五件实事工程”加快推进，新建农村公路470公里，疏浚县乡河道2900万方。新建“一池三改”户用沼气池1.56万个、无害化卫生户厕7.3万座，新增农村饮用安全水20万人。全面推动区域供水工作，覆盖范围不断扩大。培训农村实用人才和农技人员29万人次、劳动力10.1万人，新增转移7.1万人。新型农村合作医疗覆盖率99.8%。新创全面小康村180个、新农村示范村33个。

城市功能不断完善。深化“一体两翼”战略规划的研究编制，开展新一轮城市总体规划（2009-2020）修编。完成城市建设投资125亿元。新改建润扬北路、沙湾路等城区主次干道22条，翻建街巷68条。加快建设第五水厂、城北客运总站等项目。新辟公交线路4条，新增公交车200辆。建成万花园二期和润扬森林公园二期工程。整治古运河沿线环境。城区50平方公里主要区域实现无线宽带网络覆盖。建成数字化城管二期工程，道路保洁、绿化养护等方面市场化运作又有新成效。

生态市建设持续推进。新建六圩污水处理厂二期、赵庄垃圾焚烧发电厂等项目。新铺设污水管166公里。城市生活污水集中处理率84.3%。11个乡镇建成污水集中处理设施，15个乡镇通过全国环境优美乡镇命名或考核。大力开展秸秆禁烧和综合利用工作。新增造林15.3万亩，其中成片造林10.2万亩。森林覆盖率达18.1%。市区新增绿化面积160万平方米。强化对135家重点污染源企业的监管，完成年度强制性清洁生产项目。化学需氧量、二氧化硫排放量分别下降5.3%和6.9%。

四、和谐社会建设取得新成绩

民生工程深入实施。城镇新增就业7.3万人，期末城镇登记失业率2.91%。城镇职工基本养老保险参保率96.5%，新型农村社会养老保险参保率65.2%。城镇职工和居民基本医疗参保率分别达96%和95%。市区被征地农民基本生活保障“即征即保”率100%。农村五保集中供养率80%。实施市区70岁以上“三无”老人生活补贴政策。新发社会保障·市民卡3万张。市区新建和调剂廉租房280套，提供经济适用房1095套，发放购房政策性补贴782.9万元，公房解危6.13万平方米。新增住房公积金缴存职工4.6万人，归集住房公积金20亿元。提高了低保标准。落实残疾人帮扶政策。积极开展临时救助工作。关心发展慈善事业。支援四川地震灾区恢复重建任务按计划实施。市区为民办实事项目全面完成。邗江、广陵、维扬通过小康社会达标验收。

社会事业协调发展。教育教学质量进一步提升，邗江、广陵通过区域教育现代化省级验收。职业教育服务发展能力增强。特殊教育成效明显。宏志班教育覆盖所有县（市、区）。启动中小学校舍安全工程。义务教育阶段教师绩效工资稳妥实施。文化博览城建设加快推进。完成东关街二期工程，复建宋大城东门，长乐客栈对外营业。修缮阮元家庙祠堂、冬荣园、小盘谷等一批文保建筑。宋夹城遗址被授予“国家考古遗址保护公园”称号。开展第三次全国文物普查。雕版印刷、剪纸技艺入选“人类非物质文化遗产代表作名录”。扬州被评为“中国传统工艺美术特色基地”。新建社区文化活动室65个、农家书屋366个。扬剧《县长与老板》获第十一届中国戏剧节“优秀剧目奖”。成功举办第三届世界运河名城博览会，“世界运河历史文化城市合作组织”在扬成立。顺利承办第五届中日韩文化交流论坛。积极做好甲型H1N1流感防治工作。城乡社区卫生服务机构覆盖率分别为100%和97%。举办了第四届鉴真国际马拉松（半程）赛等赛事。扬州籍运动员在第十一届全国运动会上获金牌6枚。我市荣获全国“全民健身优秀组织奖”。人口计生优质服务体系覆盖率90%。有线电视入户率90%以上，市区双向数字电视技术平台通过国家验收。行政村实现宽带网络全覆盖。实施全国第二次经济普查。档案（方志）、社科研究和地震、民族宗教等工作扎实开展。

社会保持和谐稳定。社会预警和突发公共事件应急处置机制进一步健全，社会治安防控体系建设得到加强，公众安全感连续7年保持在96%以上。做好人民来信来访工作。社会矛盾纠纷大调解机制进一步完善。积极开展文明城市创建活动。开办“市民论谈”，“开放、创新、精致、优雅”的市民精神形成广泛共识。社区建设进一步加强，广陵、维扬荣获“全国和谐社区建设示范城区”称号。未成年人思想道德建设和关心下一代、老龄工作取得新进展。开展安全生产执法、治理、宣教三项行动，推进企业安全生产主体责任的落实。市场物价、食品药品监管等工作得到加强。强化国防后备力量建设，积极做好双拥和人民防空工作，荣获全国“全民国防教育先进单位”称号。

五、行政效能不断提高

行政管理体制进一步优化。完成市级政府机构改革方案并获省批准。机构编制管理进一步加强。深入开展“机关管理促进年”活动，资产、财务、工程项目、土地管理和行政收费行为进一步规范。建立以绩效考核为主要内容的考评机制。加强“部门权力内控体系”建设，建成市电子监察系统。

依法行政得到加强。制定了《扬州市2009—2014年依法行政工作规划》。积极开展行政权力清理审核和行政复议层级监督等工作，发布规范性文件20件，受理行政复议案件176件。执法规范化建设深入推进。自觉接受市人大法律监督、工作监督和市政协民主监督，办理人大代表议案、建议299件，政协提案366件。听取各民主党派、工商联和社会各界的意见和建议。深入开展法制宣传教育，我市被评为“五五”普法中期全国先进单位。积极发挥监察、审计等内部监督的作用。加强对中央扩内需保增长政策措施落实情况的监督检查。认真落实党风廉政建设责任制，抓好重点领域、重点行业的纠风和反腐败工作，查处了一批违纪违法案件。

公共服务水平不断提升。全面推进政务公开，政府新闻发布工作得到加强。制定优化投资环境、减轻企业负担等政策，开展服务经济、服务企业、服务基层“三服务”活动。深入推进行政审批“两集中、两到位”，试行“一窗式”并联审批，有效压缩投资项目办理时限。加强公共财政建设，新增财力重点用于改善民生、优化公共服务。多渠道倾听民声民意，积极解决难点、热点问题，政府公开电话和“寄语市长”回复率均在90%以上。

各位代表，回顾2009年的工作，盘点2009年的成绩，我们深深感到，在本世纪以来遇到困难最多、最大的这个年份，我们不仅较好地完成了全年目标任务，而且在许多方面有新的进展和新的突破，并为今后的发展积累了经验，奠定了基础。成绩来之不易，经验弥足珍贵，信心尤为重要。这是中共扬州市委正确领导的结果，是市人大、市政协有效监督和大力支持的结果，是全市人民同心协力、克难奋进的结果。在此，我谨代表市人民政府，向全市人民，向各位人大代表和政协委员，向所有关心、支持、参与扬州建设和发展的同志们、朋友们表示衷心的感谢和崇高的敬意!

在充分肯定成绩的同时，我们也清醒地认识到：经济回升的基础还不稳固，经济转型升级的任务还很艰巨；工业大项目、大企业不多；县域经济综合实力不强；提升城市功能与品质、推进城乡统筹还有大量工作要做；社会保障水平还需进一步提高；政府建设和公共服务与群众期望相比仍然存在差距。这些矛盾和问题都需要我们在今后的工作中，采取更加有力、更加有效的措施，切实加以解决。

2010年政府工作任务

2010年是我市总体上全面建成小康社会的决胜之年，是圆满完成“十一五”规划的收官之年，也是在发展中促转变、在创新中谋发展，为“十二五”规划启动实施创造条件的奠基之年。政府工作总体要求是：全面贯彻党的十七大、十七届三中、四中全会和中央、全省经济工作会议精神，以邓小平理论和“三个代表”重要思想为指导，深入贯彻落实科学发展观，围绕市委五届八次全会提出的“发展创新型经济、建设创新型城市”目标，坚持求发展与调结构并举，着力转变发展方式；坚持发展规模与提高质量并举，着力提升经济效益；坚持投资消费拉动与开放创新驱动并举，着力增强发展活力；坚持突出重点与统筹发展并举，着力提高城乡建设水平；坚持保增长与保民生并举，着力改善人民群众生活；坚持社会管理与政府建设并举，着力提高公共服务能力，不断开创我市经济社会又好又快发展的新局面。

全市经济和社会发展的预期目标为：地区生产总值增长13%。财政总收入增长16%，其中一般预算收入增长18%。全社会固定资产投资增长25%。研发经费支出占地区生产总值比重1.8%以上。社会消费品零售总额增长16%。城市居民人均可支配收入增长12%左右。农民人均纯收入增长10%左右。城镇登记失业率控制在4%以内。约束性指标为：单位地区生产总值综合能耗下降4.2%，二氧化硫排放量削减2.4%，化学需氧量削减5.5%。全市总体上建成全面小康社会。

重点抓好六项工作：

一、推动经济持续快速发展，进一步增强综合实力

强势推进工业经济。坚持新型工业化第一方略，启动实施“八大产业振兴发展三年行动计划”。加快主导产业优化升级。石油化工着力发展烯烃产业链、精细化工和生物化工；汽车船舶重点开发新能源汽车和特种船舶；机械装备主攻数控机床、特种钢制管和液压件。大力发展新兴产业。新能源全力推进高纯硅材料、太阳能电池和光伏发电系统集成等关键技术突破；新光源大力推进MOCVD等关键设备引进应用和外延片产业化，加快大功率、高亮度LED芯片、LED终端显示应用产品的技术研发；智能电网重点发展输配电设备及线缆、用户终端设备及器件等。加快提升传统产业和生产性服务业。进一步推进中小企业“千企升级”工程，促进企业技术、产品、管理、机制等创新。实施“培大培强”计划，新增年产销过50亿元企业3家，其中100亿元企业1家。加大对工业经济引导支持力度。设立产业发展基金，扩大对工业经济的政府引导资金规模，强化对八大产业及其重大项目、引进科技创新型领军人才的扶持与奖励。全市规模以上工业增加值增长16%。

加快振兴县域经济。制定实施县域经济新三年“争先进位、创新发展”计划，明确争先进位目标。完善县域经济发展规划，加强对重点中心镇规划和土地利用总体规划修编、产业布局和发展规划编制的指导，进一步明确产业发展重点和城市发展定位。全力实施工业强县战略，抓住世博会、长三角、沿海开发和宁镇扬区域合作的机遇，主动对接上海及苏南地区的经济转型和产业调整，承接与本地产业发展方向相吻合的先进制造业和现代服务业项目，培植优势明显的主导产业、特色产业，形成一批产值10亿元以上的龙头企业（群）。大力建设省级开发区和乡镇工业集中区，强化园区基础设施和公共服务平台建设，推动部分乡镇工业集中区与省级开发区挂联共建，新增营业收入50亿元以上乡镇工业集中区2家。积极发展建筑业，全年建筑业总产值增长12%。

突出工业大项目建设。坚持“项目为王”，以提高有效投入为目标，积极开展“项目推进年”活动，完善领导挂钩、定期督查制度，强化对大项目的服务协调、管理考核机制。狠抓龙头型、基地型、创新型大项目的引进与实施。全年新开工10亿元或1亿美元以上项目20项，工业投入增长30%。推进中海造船三期、诚德钢管三期等一批项目，加快形成百亿元生产能力。抓好亚东石化、建滔化工、九龙客车、玛切嘉利液压挺杆及缸套、力铼光电薄膜太阳能、晶澳太阳能电池片、宝胜特高压电缆、永辉特种丙纶纤维等项目建设，尽快形成50亿元以上生产能力。继续推进我市企业与央企、上市公司、跨国集团进行战略重组。加快直接融资步伐，争取1家以上企业上市。

大力发展开放型经济。围绕八大重点产业特别是新兴产业和关键领域，进一步加大招商引资、招才引智力度。突出欧美、日韩以及东南亚等地，大力推进专题招商、专业招商、产业链招商和科技合作招商，提升利用外资质态。坚持外资、民资和国资“三资联动”，加大引进央企和民资大项目力度。办好2010“烟花三月”国际经贸旅游节。全年注册外资实际到账增长20%以上。进一步推进口岸开放，提升联检机构服务水平。提高涉外企业专业化、国际化程度，促进出口品牌培育和外贸产品升级，不断优化出口结构，扩大外贸市场。规范劳务输出市场，支持有实力的企业“走出去”。外贸出口、外经营业额均增长10%以上。

积极扶持发展民营经济。进一步优化发展环境，引导民营企业练好内功，增强活力。新发展民营企业13000户以上。支持民营企业与国内500强和上市企业的合作，大力培育一批创业型、科技创新型企业。加大对“三新”科技项目、区域优势项目和特色产业项目的引进，新开工亿元以上项目100个。加强区域品牌建设，新增省级以上品牌32个。进一步放宽市场准入，扩大民间投资领域。加快发展小额贷款、融资担保、科技服务等机构，推进创业园标准厂房建设，引导和推进全民创业。全年新增民营企业注册资本金增长25%以上。

二、切实转变发展方式，进一步优化结构提高效益

着力抓好科技创新。以发展创新型经济为引领，加快产业创新、发展模式创新、体制机制创新和人才创新。继续实施“8631”行动计划，加快“八区二园”向科技创新型园区转变，完善各类公共技术服务平台功能。加大60家科技创新型大企业和300家科技成长型企业培育力度。建立健全以企业为主体的技术创新体系。新增省级以上“两站两中心”30家。继续组织开展“科技创新·产业合作”推介、“百名院士专家进百企”、“千家企业进百校”活动，启动大学科技园建设，全年实施产学研合作项目250项，新建院士工作站10家以上。研究制定与我市八大产业相关的知名高等院校和科研院所的深度合作计划。加强各类创业创新型人才的引进，突出高层次人才体系建设。制定出台《扬州市知识产权战略纲要》，推进国家知识产权试点城市建设。积极吸引市外创投、风投公司来扬投资科技创新型企业，支持发展以民间资本为主体的创投公司。新认定国家高新技术企业50家。全年高新技术产业产值增长30%。

加快提升服务业。把旅游业作为支柱产业强力发展。以蜀冈—瘦西湖风景名胜区创成国家5A级精品景区为目标，重点加强“两古一湖”等景区景点建设；抓好旅游资源整合和综合服务能力提升，促进扬子江集团、瘦西湖旅游发展有限公司等旅游龙头企业做大做强。办好第45届全国旅交会。建立旅游网站，加强境内外旅游促销。全年来扬游客和旅游总收入均增长15%以上。积极发展软件、信息服务外包等信息服务业，优化提升信息传输、信息技术服务业，推进文化创意产业发展。促进江苏信息服务产业基地（扬州）、市开发区信息系统集成和邗江嵌入式软件开发应用、维扬动漫等产业集聚区建设，全年实现软件和信息服务业销售收入80亿元。抓好长江石化、港口等一批省、市级物流园区建设，大力培育一批现代物流龙头企业和成长型企业。引导33家大企业实施非核心业务分离试点。进一步完善金融服务体系，努力增加信贷投入，优化信贷结构。新增1家以上域外金融机构，筹建市农村商业银行和再担保公司。认真落实国家扩大消费的政策措施，加快提升传统商贸业。加强核心商圈和专业市场建设，形成百亿元市场群2-3个。积极实施“万村千乡”市场工程，扩大品牌连锁经营，活跃城乡市场。优化土地供给机制，增加普通商品住房供给，支持居民自住购房需求。加大对房地产市场监管力度，保持房地产业持续平稳健康发展。

深入推进集中集约发展和节能降耗。有计划推进企业退城进园工作。加快工业产业向园区集中、高新技术产业向高新园区集中、新型产业向专业园区集中。实施LED城市照明示范工程，新建和改造LED市政照明灯具5万盏。推进太阳能屋顶计划项目建设和光伏发电并网工作。开展公共机构能耗分项计量和建筑节能改造工作。实施扬农化工等10项重点节能改造项目，培育100家节能示范企业。抓好1个国家级和12个省级循环经济试点，组织实施20项循环经济示范项目。

三、坚持统筹发展，进一步推进城乡一体化进程

构建城乡发展新格局。统筹城镇布局和产业衔接，率先完成市区1100平方公里范围内的城乡统筹发展规划，80%的乡镇完成镇区近期建设用地控制性详规编制，合理安排城乡用地、交通、水利等基础设施和市政设施等空间布局。促进公共资源与服务向农村延伸，广陵、维扬、市开发区实现城乡低保标准一体化。统筹城乡社区卫生体系建设。便民服务中心实现行政村全覆盖。加快推进城镇化步伐，研究制定农村转移人口落户城镇的政策。制订农村土地市场流转的意见。开展“双置换”改革试点。基本完成林权制度改革。推进城乡金融服务一体化。积极发展村镇银行、小额贷款公司等新型农村金融机构，有条件的乡镇组建农业担保公司。

加快推进现代高效农业。落实强农惠农政策，夯实“三农”发展基础。以增产增效增收为目标，稳定主要农产品生产。新增高效农（渔）业园区面积20万亩，其中设施农（渔）业8万亩；农业适度规模经营30万亩。创建20个万亩粮油高产示范方和20个千亩设施农业示范基地。以工业化的思路发展农业，推进更多的农产品以标准化方式进入超市。打造一批高效农业特色乡镇和专业村，培植10个10亿元以上优势主导产业。加快海峡两岸（扬州）农业合作试验区建设，新引进项目200个以上。推进农业科技创新和综合开发。加快推广水稻种植全程机械化。农产品质量安全合格率达95%以上。

稳步增加农民收入。大力发展农产品加工，加快产业化经营步伐，市级以上龙头企业销售收入增长20%，参与农业产业化经营的农户65万户。健全农业社会化服务体系。深入开展农民专业合作社示范创建活动，新组建各类合作组织500个，提高合作社的运作能力和经济效益。加大农村实用人才培训力度，转移农村劳动力4万人。依靠城镇化为农民增收提供更多的机会，增加农民工资性、财产性收入。

改善农村生产生活条件。抓好河、塘疏浚整治，完成中小型水库除险加固任务。建立农村公路、河道、村庄保洁与绿化植树的长效管护机制，农村生活垃圾集中处理率达90%。新建农村公路200公里、桥梁100座。加强秸秆综合利用，力争在全省率先实施秸秆机械化全量还田。新建“一池三改”户用沼气池1万个，完成改厕6万座。新增全面小康村100个以上、新农村示范村30个以上。

四、着力优化城市功能，进一步提升城市品质

全面推进重大基础设施建设。加快沪陕高速江六段、宁启铁路复线及电气化改造、扬州港5号泊位和乌塔沟分洪道等重点工程建设，确保京杭运河扬州段“三改二”工程竣工，建成江海高速扬州段、安大公路宝应段。开工建设苏中江都机场、沪陕高速江广段扩容、淮河入江水道整治、高水河工程和500千伏扬州西输变电工程。进一步推进连淮扬镇铁路、京沪高速扩容及南延、扬天高速公路、江都港区新泊位、仪征港区液体化工码头二期和扬州二电厂三期等项目前期工作。

加快主城区建设。围绕迎接扬州建城2500周年，按照“精当规划、精致建设、精细管理”的要求，进一步提升城市品质。完成城市总体规划的修编工作。完善主城交通网络，延伸改造北环路、西三环等道路，打通城区环路；实施文昌东路东延、新328国道、扬仪路等工程。不断完善城市供水、供气、污水处理等公用设施。开工建设会展中心二期、规划馆、运博会永久性会址等工程，加快建设赵庄垃圾焚烧发电厂。积极保护老城区，优化改造旧城区，不断完善新城西区，加快广陵新城、南部临港新城、蜀冈生态新区、蒋王等片区建设。继续整修东关街历史文化街区，实施丁氏、马氏住宅群维修等工程，整治甘泉路、广陵路，修缮传统民居100户。完善数字化城管体系，扩大城市管理市场化运作范围，健全长效管理机制。

积极创建全国文明城市、国家生态市和森林城市。深入开展全国文明城市创建工作，重基础、重过程、重长效，不断提升市民素质，切实提高人民群众生活的满意度和幸福感。优化环境监管体系，对75家重点排污企业实行视频监控。加大对饮用水源地整治保护力度，县级以上集中式饮用水源地实现水质自动监控。全面完成淮河流域扬州段水污染防治任务。加快污水管网建设，建成六圩污水处理厂二期工程。全年空气优良天数320天以上。创成全国环境优美乡镇15个、省市级生态村50个。突出森林生态建设，优化城市大型生态绿地布局，加强永久性绿地保护，大力推进城区、园区、企业绿化工程，做好现有骨干道路、城市出入口、河道两侧的绿化提质增量，继续高标准建设大江风光带和一批公共绿地，市区新增绿化面积100万平方米以上。全市新增造林15万亩以上，森林覆盖率提高1.5个百分点。全面实现“十一五”化学需氧量和二氧化硫排放量削减目标。

五、注重改善民生，进一步促进基本公共服务均等化

努力促进就业创业。深入实施积极的就业政策，全年采集就业岗位9.5万个，新增城镇就业4.6万人。完善城镇“零就业家庭”和农村“零转移家庭”就业援助长效机制，重点推进高校毕业生、就业困难人员和农民工就业，建成充分就业社区80个。加强创业服务平台建设，建成创业孵化基地15个。推介创业项目200个，扶持自主创业1000人，带动就业6000人。强化职业技能培训和公共就业服务，全年培训城乡劳动者7.6万人，其中就业再就业培训3.6万人。

完善社会保障体系。全年城镇职工基本养老保险净增3.2万人，新型农村社会养老保险参保率达80%，市区被征地农民基本生活保障“即征即保”率稳定在100%。稳步推进医药卫生体制改革，加强城乡居民公共卫生服务体系、基本医疗服务体系、基本医疗保障体系及基本药物制度建设，推进市二院公立医院改革试点。发展惠民医疗服务。城镇职工和城镇居民基本医疗参保率均达98%。大学生参保率100%。全市新型农村合作医疗覆盖率稳定在97%以上，人均筹资标准不低于150元。发放社会保障·市民卡20万张。推进建立居民电子健康档案。抓好住房公积金归集扩面工作。进一步完善廉租房和经济适用房保障制度，努力提高对住房困难家庭的保障水平。完成农村扶贫三年目标。

做好社会福利和救助工作。完善城乡低保、农村五保供养标准自然增长机制。推进残疾人保障与服务体系建设，强化残疾人康复服务。免费培训1600名残疾人，帮助1000名残疾人实现就业。建设50个县级残疾人扶贫基地。加快推进无障碍城市创建。进一步健全临时救助体系，建成市综合性救助中心。对市区城市低保对象实行常见病常用药品资助制度。加大低保边缘对象的临时救助力度。做好支援四川灾区九龙镇重建工作。

集中力量办好民生大事。推进基本公共服务均等化，为城乡居民免费实施9大类22项基本公共卫生服务，打造“15分钟健康圈”。加快区域供水工程建设，建成第五水厂一期工程，区域供水覆盖率市区和仪征100%、高邮和江都达70%以上、宝应达60%以上。新增73万人饮用安全水。突出抓好市区“五改一解”，改造老小区11个、老城区背街小巷50条、城中村10处、农贸市场10个，改扩建一批老城区绿地，实施公房解危6万平方米，让老城区市民享受到更好的公共服务与居住环境。大力度改善公共交通，市区新增公交车400辆、更新出租车100辆，新建公交首末站和停车场6个。

六、维护社会繁荣稳定，进一步促进社会和谐

发展各项社会事业。均衡发展义务教育，优化发展高中教育，积极发展职业教育。改善办学条件，实施中小学校舍安全工程。继续推进区域教育现代化。实施农村留守儿童关爱工程。新招收宏志班20个。建成市新图书馆、音乐厅和美术馆等项目。启动建设青少年活动中心、妇女儿童活动中心。继续做好大运河联合申遗和扬州地方申遗。推进文化体制改革。深入开展文化下乡、进社区活动。全市数字电视用户达40万户，有线电视入户率94%以上。建设体育公园体育场，建成市游泳跳水馆。办好第五届鉴真国际马拉松（半程）赛。推进全民健身，加快体育强县（市）创建工作。人口计生优质服务体系覆盖率达100%。组织实施全国第六次人口普查。做好民族宗教工作。继续办好“市民论谈”，弘扬市民精神，提高社会主义精神文明建设水平。

努力建设和谐社区。建立健全“四位一体”的社区管理服务体制机制。规范社区居民自治，大力发展适应居民需求的社区医疗卫生、劳动就业、文化体育、养老等服务，全市新建社区居家养老服务中心（站）120个，升级150个。整体联动，全方位推进农村社区建设。加强社区工作者队伍建设，合理配备社区专职工作者，力争做到一个社区有一名任职大学生。建立健全社区自我服务与管理制度，引导和支持社会组织参与社区管理。开展和谐社区示范单位创建活动，城区新建15个社区服务中心和邻里中心。

保持社会稳定。以社会矛盾化解、社会管理创新、公正廉洁执法为重点，积极打造“平安扬州”“法治扬州”。加强社会治安综合治理基层基础工作，健全社会治安防控体系，积极防范和依法打击各类违法犯罪活动。完善应急管理体系，增强突发性公共事件应急处置能力。切实做好社会稳定风险评估工作。严格落实安全生产责任制，推行安全生产专家工作制度，强化对危险化学品、船舶制造、道路交通和食品药品等领域的安全监管，严防重特大事故发生。进一步提高防灾抗灾能力。加强市场监管，保持物价稳定，继续抓好放心消费工程。落实信访工作领导责任制，认真解决各类信访突出问题。大力加强法律援助工作，维护群众合法权益。完善国防动员体制机制，深入开展双拥和国防教育，巩固发展军政、军民团结。

努力开创政府工作新局面

形势的新变化、发展的新任务，要求我们进一步坚持科学发展观，突出以人为本的理念，强化开拓创新的意识，增强公共服务的水平，切实加快职能转变，推进管理创新，努力建设人民满意的政府。

增强忧患意识，提高争先进位的责任感。面对改革发展的任务和区域竞争的压力，我们将进一步居安思危、居安思进，聚焦聚力抓经济，克难求进谋发展，推动经济尤其是工业经济做强做大；进一步增强大局意识、整体观念，自觉服从大局，主动服务大局，做到心往一处想、劲往一处使，提高为科学发展服务的本领；进一步在全省工作中找准部门定位，不断创新创优，加大向上争取政策、资金、项目的力度，以服务发展的业绩论贡献，以在全省同行中的位次比高低；进一步奋发有为、狠抓落实，把困难估计得更充分，把措施考虑得更周全，面对各种任务和挑战，拉得出、打得响、干得成。

重视学习调研，提高推动发展、服务基层的能力。按照“眼界宽、思路宽、胸襟宽”的要求，深入开展创建学习型机关活动，抓好“新知学堂”讲座、读书会等各类公务员培训工作，不断提高综合思维能力、专业化操作本领。加强调查研究，以“发展创新型经济、建设创新型城市”为目标，做好“十二五”规划编制工作，重点开展生产力空间布局、转型发展方向、重大基础设施建设、社会事业协调发展、保障和改善民生等重要内容的研究，特别是紧盯国际先进制造业、服务业发展趋势和科技创新领域的新成果，牢牢把握国家产业政策新变化和区域经济发展的新要求，加强我市战略性新兴产业发展的研究，抢抓新一轮发展的制高点。着力破解发展难题，帮助企业解决资金、土地、劳动力和能源供应等问题，打造良好的发展环境。

深化作风建设，提高依法行政水平。自觉接受市人大及其常委会法律监督和工作监督，积极支持市政协履行政治协商、民主监督、参政议政职能，主动听取各民主党派、工商联、人民团体的意见和建议。认真办理人大代表议案、建议和政协提案。重视群众监督和舆论监督。办好政府公开电话、“寄语市长”和部门服务热线。广泛开展法治城市创建，认真落实行政执法责任制，规范行政执法自由裁量权，强化行政执法监督和行政复议工作。全面完成“五五”普法任务。激励广大公务员敢于负责、勇于担当，在服务大局中找准工作的着力点，做到工作思路项目化、操作方案专业化，工作任务可定义、可量化、可操作、可考核、可追究，切实提高工作成效。坚持深入一线、深入基层，问政于民、问需于民、问计于民，凝聚民心，集中民智，引导广大群众积极投身到创业创新创优中来。加大治懒治庸治浮力度，务实作风，从严考核，通过督查会、会办会、现场观摩会等形式，面对面、硬碰硬、实打实地推进各项工作有序、有力地开展。

切实转变职能，创新政府工作。认真实施新一轮政府机构改革。加强公共财政建设。不断扩大税源，提升税收服务质量；推进财税科学化、精细化管理；优化财政收支结构，从严控制一般性支出。做好国有资产经营与管理工作。深化行政审批制度改革，优化办事流程，提高办结效率。建立重大行政决策调研制度，完善决策信息和智力支持系统。深入推进行政权力网上公开透明运行，坚持政府新闻发布和重要行政事项、公共政策社会公示制度。强化资源整合、政策集成和协调沟通，形成工作合力，提高政府总体效能和系统解决问题的能力。

加强反腐倡廉，建设廉洁政府。进一步落实党风廉政建设责任制，深入开展反腐倡廉教育，加快推进惩治和预防腐败体系建设。坚持用制度管权、管事、管人，加强对财政资金、重大投资项目和民生事项的审计，深入开展治理商业贿赂和工程建设领域突出问题等专项工作，严肃查处违法违纪行为。深入开展创建“规范收费学校、规范收费医院”活动，坚决纠正损害群众利益的不正之风。大力倡导艰苦奋斗、勤俭办事，深化节约型机关建设。

各位代表！站在历史的新起点，面对人民的新期待，我们深感责任重大、使命光荣。让我们在中共扬州市委的领导下，紧紧依靠全市人民，同心同德，扎实工作，奋发进取，为不折不扣地完成2010年的各项任务，为加快实现全面小康和“十一五”规划目标，为建设“创新扬州、精致扬州、幸福扬州”而努力奋斗！
